# Misdiagnosis of rectal misplaced glands as rectal cancer: A rare case report

**DOI:** 10.1097/MD.0000000000042786

**Published:** 2025-06-20

**Authors:** Weitao Huang, Xiaowei Han, Guozheng Zhang

**Affiliations:** a Department of Radiology, The Quzhou Affiliated Hospital of Wenzhou Medical University, Quzhou People’s Hospital, Quzhou, China.

**Keywords:** chronic rectal inflammation, misdiagnosis, misplaced glands, rectal cancer

## Abstract

**Rationale:**

We report a rare case of rectal misplaced glands (RMG) with a 2-year disease course. Digital rectal examination identified a semi-circumferential mass approximately 4 cm from the anal verge. Imaging studies revealed irregular thickening of the middle and lower rectal walls with multiple mildly enlarged surrounding lymph nodes. Colonoscopic biopsy excluded a diagnosis of rectal cancer. Upon further evaluation of the patient’s history, it was determined that chronic inflammation caused by prolonged soapstick enema use led to ectopic glandular proliferation within the rectal wall.

**Patient concerns:**

A 72-year-old female presented with a 2-year history of altered bowel habits and a 1-week history of anal fullness.

**Diagnoses:**

Imaging studies, including computed tomography, magnetic resonance imaging, and positron emission tomography–computed tomography, suggested rectal cancer. However, colonoscopic biopsy pathology revealed extensive inflammatory exudates, necrotic tissue, focal granulation tissue formation, and severe compression artifact. Considering the patient’s 20-year history of soapstick enema use, clinicians diagnosed the condition as RMG.

**Interventions:**

The patient was advised to discontinue the use of soapstick enemas and was managed with regular follow-up and observation.

**Outcomes:**

Misdiagnosis of RMG as rectal cancer could lead to unnecessary radical surgical interventions. Raising awareness of this rare condition and accurately interpreting pathological findings are critical to improving patient outcomes.

**Lessons:**

This case highlights the importance of considering rare conditions like RMG in the diagnosis of rectal masses. A detailed patient history, such as prolonged soapstick enema use, was crucial in identifying the cause. Imaging may suggest rectal cancer, but biopsy findings showing inflammation helped differentiate RMG.

## 1. Introduction

Rectal cancer was a significant global health concern characterized by abnormal cell growth within the rectum. Early detection and accurate diagnosis were critical for achieving favorable treatment outcomes.^[1]^ Various benign and malignant lesions could mimic the clinical and imaging features of rectal cancer, potentially leading to misdiagnosis. Among these, rectal misplaced glands (RMG) represented a rare condition in which ectopic glandular tissue appeared within the rectal wall.^[2]^ Differentiating this condition from malignant tumors was particularly challenging.

The presentation of RMG often resembled that of rectal cancer, with symptoms including rectal bleeding, changes in bowel habits, and the discovery of palpable masses during examination.^[3]^ Histopathological evaluation was typically essential for a definitive diagnosis; however, clinicians often initially relied on imaging studies and clinical presentations, which could result in diagnostic errors.

In this report, we described a rare case of the RMG that was misdiagnosed as rectal cancer. This case highlighted the importance of considering differential diagnoses in patients presenting with rectal symptoms and emphasized the need for tailored diagnostic approaches. Through this case report, we aimed to enhance awareness of the RMG and contribute to the existing literature by detailing the clinical, imaging, and histopathological findings of this unusual presentation. We sought to underscore the importance of meticulous and comprehensive diagnostic strategies to avoid unnecessary surgical interventions and ensure appropriate patient management.

## 2. Case presentation

**Figure 1.**
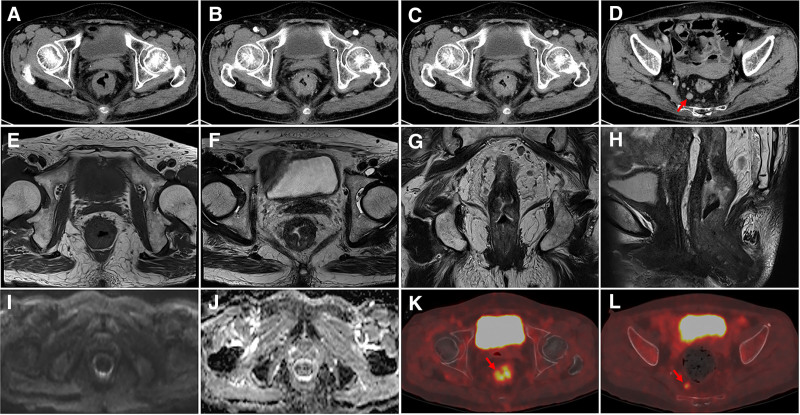
(A–D) The CT images of the patient. Enlarged lymph nodes surrounding the lesions (red arrow). (E–J) The MRI images of the patient. (K, L) The PET-CT images of the patient. the rectal lesion (K, red arrow) and enlarged lymph nodes (L, red arrow). CT = computed tomography, MRI = magnetic resonance imaging, PET-CT = positron emission tomography-computed tomography.

**Figure 2.**
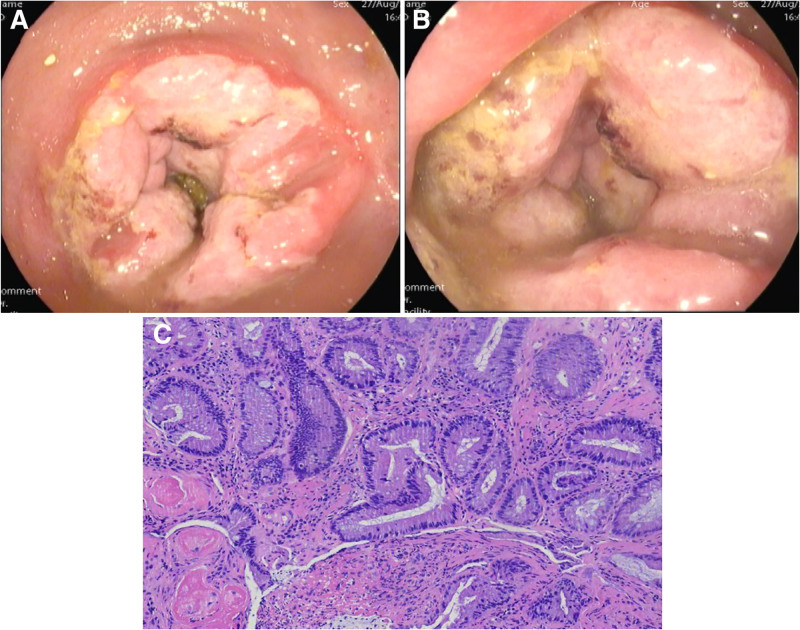
(A, B) The colonoscopic images obtained from the patient. (C) Image of the pathology report (H&E, ×200).

A 72-year-old female patient presented with a 2-year history of altered bowel habits, characterized by increased defecation frequency, reduced stool volume, tenesmus, and lower abdominal distension without abdominal pain, nausea, vomiting, or reflux. The symptoms were initially left untreated. One week prior to admission, the patient reported a sensation of anal fullness and sought medical evaluation. Digital rectal examination revealed a semi-circumferential, fragile, poorly mobile mass approximately 4 cm from the anal verge, without tenderness or blood-stained gloves. Enhanced CT and MRI imaging (Fig. 1A–J) demonstrated irregular thickening of the middle and lower rectal walls with multiple mildly enlarged surrounding lymph nodes, suggesting possible rectal cancer. Positron emission tomography-computed tomography (Fig. 1K, L) showed annular, irregular thickening of the rectal and anal canal walls with increased FDG uptake (SUVmax ~7.96). The serosal surface was mildly irregular, with blurring of the surrounding fat planes. Multiple mildly enlarged perirectal lymph nodes (maximum diameter ~1.1 cm) exhibited slightly increased FDG uptake (SUVmax ~3.57), supporting a diagnosis of possible rectal malignancy. Colonoscopy (Fig. 2A, B) revealed circumferential thickening of the lower rectal mucosa, appearing slightly pale. Biopsy showed medium-textured tissue. Pathology (Fig. 2C) identified colonic mucosa with crypt dilatation and mucin deposition, stromal fibrosis, smooth muscle proliferation, capillary proliferation, ulceration, extensive inflammatory exudate, necrotic tissue, focal granulation tissue formation, and significant compression artifact. Upon detailed evaluation, including the patient’s history of 20 years of soapstick enema usage prior to defecation, chronic rectal inflammation due to enema-induced injury was identified as the underlying cause. Glandular misplacement was attributed to chronic inflammation, particularly during periods of mucosal barrier disruption and repair. The diagnosis of rectal glandular heterotopia secondary to long-term enema use was confirmed. The patient was advised to discontinue the use of soapstick enemas and was managed through regular follow-up and observation. The patient did not return to our hospital for follow-up after discontinuing the use of soapstick enemas prior to the submission of this manuscript, and thus no information on symptom changes or relevant examination results during the follow-up period could be obtained.

## 3. Discussion

RMG, also known as misplaced or ectopic glands, represent a rare and often overlooked pathological phenomenon. These structures originate from the abnormal localization of glandular tissue, which may occur during embryonic development or secondary to inflammatory or traumatic processes.^[4]^ Although inherently benign, the striking resemblance of RMG to rectal adenocarcinoma on imaging and endoscopic evaluation poses significant diagnostic challenges. This case underscores the importance of distinguishing RMG from rectal cancer to avoid misdiagnosis and the associated psychological and therapeutic consequences.

The pathological overlap between RMG and rectal adenocarcinoma lies primarily in shared features such as glandular crowding, structural irregularity, and occasional stromal reactions. However, critical distinctions exist; RMG typically lack hallmark characteristics of adenocarcinoma, including nuclear atypia, pleomorphism, and heightened mitotic activity.^[5,6]^ In this case, although glandular crowding and structural irregularity initially raised suspicion for malignancy, the absence of nuclear atypia served as a pivotal clue supporting the diagnosis of RMG.

Diagnosing RMG requires an integrative approach combining pathology with clinical and imaging findings. During endoscopic evaluation, invasive rectal cancer often manifests as a mass, irregular mucosa, or ulceration, while RMG rarely cause prominent endoscopic abnormalities, although they may occasionally be associated with polyps or mucosal scarring.^[7]^ Imaging modalities such as CT and MRI can aid in assessing local invasion, lymphadenopathy, or metastatic disease, which are hallmarks of malignancy but typically absent in RMG.^[5]^

Misdiagnosis of RMG as rectal cancer can lead to unnecessary interventions, including major surgical resections, chemotherapy, and radiotherapy, along with their associated complications. This case highlights the importance of a multidisciplinary approach involving pathologists, radiologists, and clinicians to achieve an accurate diagnosis and avoid overtreatment. In the present case, an initial diagnosis of rectal adenocarcinoma was revised following pathological reevaluation, thereby sparing the patient from unnecessary invasive therapies.

RMG are exceedingly rare, and reports of misdiagnosis are limited in the literature. A review of the literature reveals only a handful of cases describing similar diagnostic challenges. The rarity of this condition underscores the importance of increasing awareness among clinicians and pathologists. Furthermore, comprehensive documentation of cases like this 1 contributes to the development of diagnostic guidelines, thereby improving patient outcomes.

This rare case of RMG misdiagnosed as rectal cancer emphasizes the diagnostic complexity in differentiating benign from malignant rectal glandular lesions. A thorough understanding of pathological features, combined with correlation to clinical history, is critical to preventing misdiagnosis and overtreatment. Future research and case reports are needed to deepen the understanding of RMG and their clinical significance, ultimately enhancing diagnostic accuracy and patient care.

## Author contributions

**Conceptualization:** Weitao Huang.

**Data curation:** Weitao Huang.

**Formal analysis:** Weitao Huang.

**Funding acquisition:** Xiaowei Han, Guozheng Zhang.

**Investigation:** Weitao Huang.

**Methodology:** Weitao Huang.

**Project administration:** Weitao Huang.

**Visualization:** Guozheng Zhang.

**Writing – original draft:** Weitao Huang.

**Writing – review & editing:** Guozheng Zhang.
